# Highly selective detection of individual nuclear spins with rotary echo on an electron spin probe

**DOI:** 10.1038/srep15402

**Published:** 2015-10-26

**Authors:** V. V. Mkhitaryan, F. Jelezko, V. V. Dobrovitski

**Affiliations:** 1 Ames Laboratory US DOE, Ames, Iowa, 50011, USA; 2 University of Ulm, Institute of Quantum Optics and Center for Integrated Quantum Science and Technology, 89081 Ulm, Germany

## Abstract

We consider an electronic spin, such as a nitrogen-vacancy center in diamond, weakly coupled to a large number of nuclear spins, and subjected to the Rabi driving with a periodically alternating phase. We show that by switching the driving phase synchronously with the precession of a given nuclear spin, the interaction to this spin is selectively enhanced, while the rest of the bath remains decoupled. The enhancement is of resonant character. The key feature of the suggested scheme is that the width of the resonance is adjustable, and can be greatly decreased by increasing the driving strength. Thus, the resonance can be significantly narrowed, by a factor of 10–100 in comparison with the existing detection methods. Significant improvement in selectivity is explained analytically and confirmed by direct numerical many-spin simulations. The method can be applied to a wide range of solid-state systems.

Detecting a single nuclear spin is an ultimate goal in nuclear magnetic resonance, actively pursued using various techniques^1^,^2^,^3^,^4^,^5^,^6^,^7^,^8^. Moreover, one-by-one detection, characterization, and manipulation of individual nuclear spins in solids is vital for harnessing them as a resource for quantum information processing^9^,^10^,^11^,^12^,^13^,^14^,^15^,^16^,^17^,^18^,^19^,^20^. The crucial problem, hindering the progress in this area, is to separate the weak signal, produced by a given weakly coupled nuclear spin, from the much stronger background created by all other spins and by the ambient magnetic noise. I.e., the detector must possess high selectivity to separate the signal of the target spin from the action of other spins and from magnetic noise.

It has been recently shown that an electron spin of the nitrogen-vacancy (NV) centers in diamond is an excellent nuclear spin detector^5^,^6^,^7^,^8^,^21^,^22^,^23^,^24^,^25^,^26^,^27^. By applying the dynamical decoupling protocols to the NV spin (pulse^5^,^6^,^7^ or continuous^8^,^27^), the individual nuclear spins around the NV center can be detected one by one in a resonant manner: the NV spin becomes decoupled from all nuclear spins, except the one which satisfies a stringent resonance condition. The resonating nuclear spin strongly affects the NV spin motion, and hence can be detected, and the width of the resonances produced by different nuclear spins determines selectivity. However, in all current schemes this width is naturally limited, being determined by the strength of the hyperfine coupling between the target nuclear spin and the NV spin.

Overcoming this limitation, and drastically narrowing the resonances in a systematic way, in order to significantly improve the detection selectivity, is of much importance. Here we present a scheme which achieves that goal. It employs the periodically changing Rabi driving on the NV center’s spin (multiple rotary echo)^28^,^29^,^30^ with a specially chosen period to detect the nuclear spins. The width of a resonance, corresponding to detection of a given nuclear spin is inversely proportional to the Rabi driving strength, and therefore can be directly adjusted in experiment. As a result, the resonance peak width is narrowed by a factor of 10 − 100 in comparison with the existing schemes. This narrowing greatly enhances the resolution, which is very important for accurate characterization of the nuclear-spin environment of the NV center^6^,^7^,^8^,^12^,^13^ and for NV-based nuclear magnetic resonance at nanoscale^21^,^25^,^26^. Thus, our approach combines excellent protection from magnetic noise offered by the rotary echo, and greatly enhanced selectivity in detection of individual nuclear spins. This approach can also be used with other electronic spins as detectors, e.g. spins of dopant atoms in semiconductors or other defect centers in various materials.

## Results

**Figure 1:**
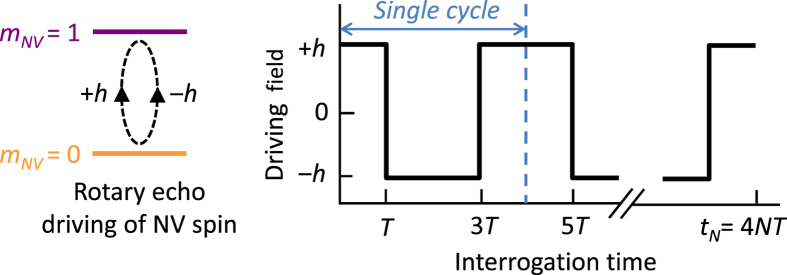
Rotary echo protocol: Rabi driving is applied to the NV electron spin at the frequency of the transition between the states *m*_*NV*_ = 0 and *m*_*NV*_ = 1. The phase of driving is periodically switched by 180°, i.e. the driving field in the rotating frame periodically changes between +*h* and −*h*.

We consider the electronic spin *S* = 1 of the NV center subjected to a moderate (tens to hundreds of Gauss) static bias field along the symmetry axis. The NV spin possesses three well-separated states *m*_*NV*_ = 0, 1 and −1 (denoted below as 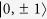), and is manipulated by applying the microwave driving at resonance with the transition between the states 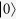 and 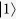, as shown in Fig. 1; the level 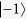 remains idle and will be further ignored. The NV spin is coupled to a nuclear spin *I*: e.g., a ^13^C nuclear spins in diamond (*I* = 1/2), coupled to the NV center via dipolar interaction. To simplify consideration, we temporarily omit the coupling of the NV spin to other ^13^C spins in the sample, considering the case of many nuclear spins later (see also Supplementary Information). In the coordinate frame rotating with the frequency of the resonant microwave driving, the system is described by the Hamiltonian


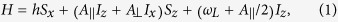


where 
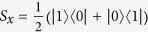
 and 
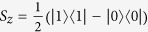
 describe the NV spin, and *I*_*x,z*_ are the operators of the target nuclear spin, *h* is the Rabi driving field (usually, few to few tens of MHz), *ω*_*L*_ is the Larmor frequency of the nuclear spin, 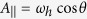 and 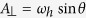 are the components of the hyperfine coupling, and we assume *ħ* = 1 throughout the text.

During the rotary echo experiment the direction of the Rabi driving field (i.e. the phase of driving) periodically changes between +*x* (along the *x*-axis) and −*x* (opposite to the *x*-axis), as shown in Fig. 1. Here we consider symmetrized protocol, consisting of *N* rotary echo cycles; within each cycle the driving field is along +*x* during the two outer segments, each of duration *T*, and is along −*x* during the inner segment of duration 2*T*. Therefore, the Hamiltonian (1) describes the two outer segments of the rotary echo cycle, and the Hamiltonian for the inner segment is obtained by replacing *h* → −*h*. Below, we denote these two Hamiltonians as *H*_+_ and *H*_−_, respectively, so the evolution operator for a single cycle is


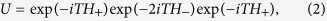


and the full *N*-cycle protocol is described by the evolution operator *U*(*N*) = *U*^*N*^. Generally, periodic switching of the Rabi field in the regime of strong driving 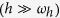 leads to highly efficient decoupling of the NV electronic spin from the surrounding spins^29^,^30^,^31^. However, in a special resonant regime, where the Rabi field is switched synchronously with the Larmor precession of the target nuclear spin, quantum states of the NV center spin and the target nuclear spin become entangled. After many rotary-echo cycles, 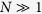, this entanglement becomes detectable despite the weak coupling between the NV and target spins. This approach is close in spirit to the “stroboscopic matching” idea^32^.

To qualitatively understand this phenomenon, we analyze the evolution of the system assuming small coupling 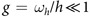, so that the zero-order Hamiltonians are 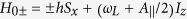. The corresponding zero-order evolution operator is given by


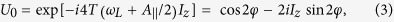


where 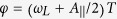. I.e., the nucleus and the NV center are practically decoupled: over one cycle, the nuclear spin rotates around the *z*-axis by the angle 2*φ*, and this rotation does not depend on the state of the NV spin. However, if the driving is switched in resonance with the nuclear spin precession, so that the switching time has a special value,


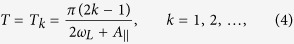


then sin2*φ* = 0, and the operator *U*_0_ is unity, i.e. the effect of the zero-order Hamiltonian is null after one cycle. Then the smaller higher-order corrections will become important, since their effect will accumulate over many rotary echo cycles, undisturbed by the zero-order terms. The detailed analysis (details are given in Supplementary Information) shows that in this resonance case the nuclear spin evolution changes drastically: the rotation axis tilts towards *y* axis, the rotation angle per cycle is of order of 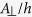, and most importantly, the nuclear spin entangles with the NV spin through the operator 
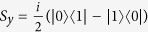
. I.e., at resonance the nuclear spin rotates in different directions depending on whether the NV spin is in the state 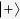 or 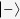, where 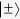 are the eigenstates of *S*_*y*_ with the eigenvalues *S*_*y*_ = ±1/2, respectively.

**Figure 2:**
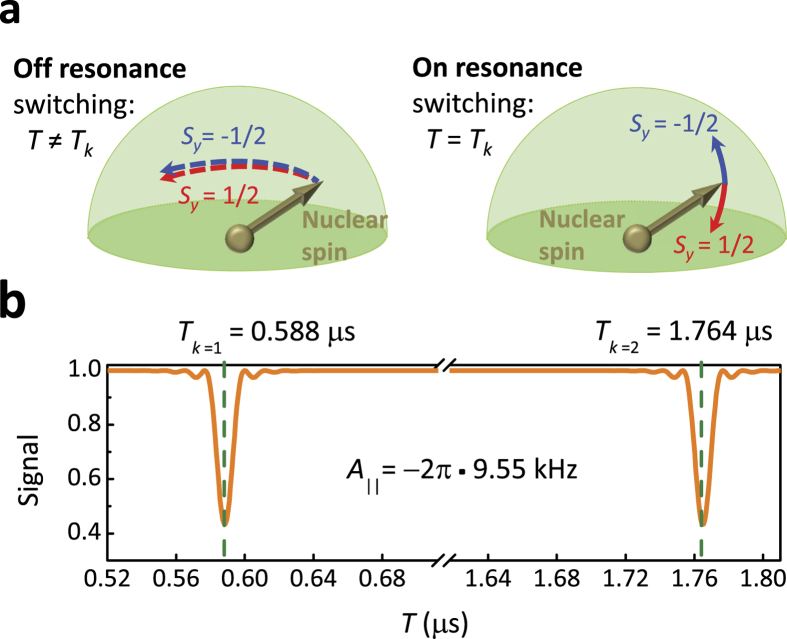
(**a**) Generally, the rotary echo protocol efficiently decouples the nuclear spin from the NV center: evolution of the nuclear spin is practically independent of the NV spin state (left). However, when the switching is on resonance with the nuclear spin precession (*T* = *T*_*k*_), the nuclear spin entangles with the NV spin: its evolution is conditioned on the initial NV spin state (right). (**b**) When *T* becomes equal to the resonance value *T*_*k*_, entanglement with the nuclear spin leads to faster decay of the NV electron spin, seen as a resonant drop of the signal. Graph shows the rotary echo signal (defined everywhere in the paper as 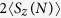) after *N* = 50 cycles as a function of *T*, for *ω*_*L*_ = 2*π* · 430 kHz, driving *h* = 2*π* · 3.51 MHz, and coupling constants 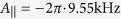, 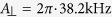. The resonances of the orders *k* = 1 and *k* = 2 are marked with green dashed lines.

We consider an experiment where the NV spin is prepared in the state *m*_*NV*_ = 1, and the operator *S*_*z*_(*N*) is measured after *N* rotary echo cycles. At resonance, the initial coherent superposition 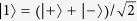 is decohered in the basis 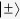 as a result of entanglement with the unpolarized nuclear spin, so that the resonance can be detected by sudden onset of strong decay of *S*_*z*_(*N*) Analytical solution, which remains valid at long times 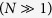, is derived as explained in the Methods section, also see Supplementary Information for details. The result is:


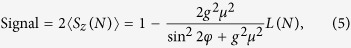


and in the vicinity of the resonance 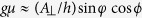 and 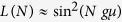, where *ϕ* = *hT*. The signal envelope as a function of the switching time *T* (or the angle *φ*) has Lorentzian shape with the width 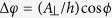 (for 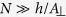) and the depth 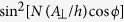. To maximize the depth, the driving should be adjusted to have *ϕ* = *hT* = *πm* with integer *m*. This also minimizes interference between different nuclear spins and the influence of the fluctuations in the driving power, see Supplementary Information, and below we always assume this condition satisfied. Typical dependence of the signal on *T* is shown in Fig. 2, where the resonances at *T*_*k*=1_ and *T*_*k*=2_ are clearly seen. The analytical solution (5) is very precise: it practically coincides with the direct numerical simulation of the two-spin system described by the Hamiltonian (1).

For a weakly coupled nucleus, located sufficiently far from the NV center, the hyperfine coupling is determined primarily by dipolar interaction, so that 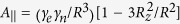, where *R* is the vector connecting the positions of the NV center and the nucleus, and *γ*_*e*_ and *γ*_*n*_ are the electronic and nuclear gyromagnetic ratios, respectively. Therefore, the nuclei from different locations produce the peaks at different values of *T*, and hence can be resolved.

**Figure 3:**
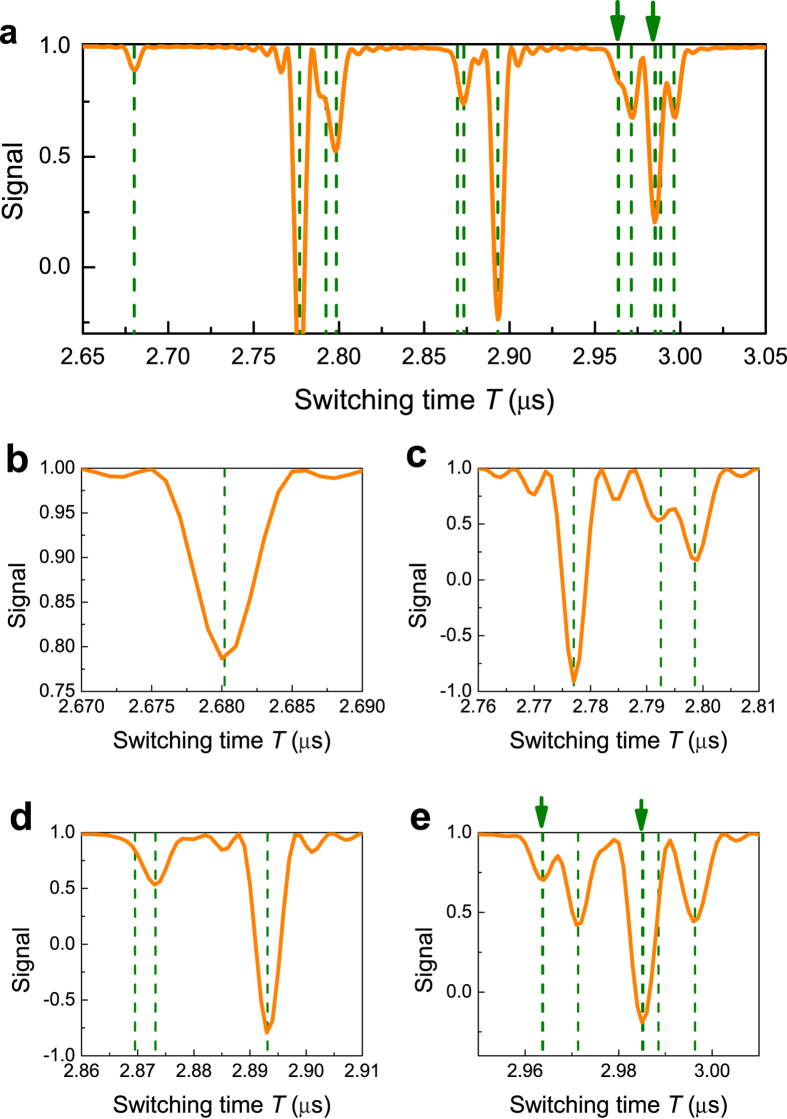
(**a**) Rotary echo signal after *N* = 100 cycles, as a function of the switching time *T* for a NV spin coupled to 14 ^13^C nuclear spins, randomly placed in the diamond lattice with natural abundance, for *ω*_*L*_ = 2*π* · 428 kHz. Driving *h* is adjusted so that *hT* = 28*π* (so that 4.59 ≤ (*h*/2*π*) ≤ 5.28 MHz). The region corresponding to the resonances of the order *k* = 3 is shown. Theoretically expected resonances are marked with green lines; each of the lines marked with arrows correspond to a pair of nuclei located at the symmetry-related sites, and having almost the same value of 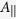. Parameters of the nuclear spins are given in Supplementary Information. (**b**–**e**) Magnified view of the different areas of the panel (**a**).

To test our approach, and to illustrate its performance under realistic circumstances, we performed direct simulations of the rotary-echo detection for the NV center coupled to 14 nuclear spins of ^13^C randomly located in diamond lattice. The simulation results, given in Fig. 3 for *k* = 3, clearly show the sharp, well-resolved peaks, corresponding to different nuclei, with selectivity in sub-kHz region, even for very modest driving *h* ~ 2*π* · 5 MHz (see also Supplementary Information for more details). Some nuclei, located at the symmetry-related positions in the lattice, have the same 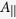 and hence the same resonance value of *T*; to resolve them, the static bias field should be tilted away from the symmetry axis of the NV center^6^. The simulations also confirm our conclusion (see Supplementary Information) that the NV-mediated interaction between the nuclear spins, caused by the driving, does not noticeably affect the detection efficiency.

For a fixed total interrogation time *t*_*N*_ = 4*NT*, the resonance width determines selectivity of the protocol, i.e. the ability to resolve two nuclei with close hyperfine couplings 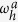 and 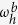 (i.e. 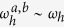, 
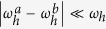
). High selectivity of the suggested scheme is determined by the small width of the resonances achieved at 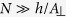, this width is 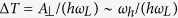 and decreases as driving *h* increases (for estimates here and below we neglect the difference between 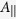 and 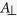, taking 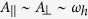). The resonance width Δ*T* should be compared to the distance between the *k*-order resonances from two nuclear spins, which is 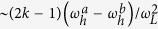. Thus, the resonances are resolved when 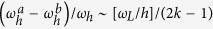. For a typical experiment, detecting ^13^C spins, with the bias field of 400 G (*ω*_*L*_ = 2*π* · 428 kHz) and driving of *h* = 2*π* · 10 MHz, for *k* = 3, the condition of resolved resonances is 
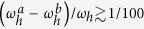
.

Note that the protocols discussed in this work are, in fact, two-dimensional, with the signal depending on some adjustable parameter (*T* for our scheme, *τ* for the pulse dynamical decoupling, etc.), and on the total interrogation time. Analyzing the latter dependence was proposed^8^ as a way to increase resolution; this option can also be used within the pulse dynamical decoupling detection, and within the protocol presented here. However, the resulting improvement strongly depends on experimental details, and is beyond the scope of the present paper.

## Discussion

The fact that the resonance width can be adjusted by simply changing the driving *h* is a key feature of the proposed protocol, which leads to great improvement in selectivity. In the currently existing detection schemes, the width of the resonance is naturally limited, being determined by the coupling constants 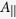 and 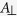. The scheme proposed here is the first, to our knowledge, where the width of the resonances can be tuned at experimentalist’s will; this allows narrowing of the resonances by a factor of 10–100 in comparison with other existing schemes (see Supplementary Information for details), albeit at the expense of the longer interrogation time. Also, stronger driving greatly improves the coherence time of the NV spin^29^,^30^,^31^. Moreover, using the rotary echo protocol with the optimal choice of driving *h* significantly reduces the impact of slow fluctuations in the driving strength, which is an important limiting factor for the Hartmann-Hahn double resonance detection^8^,^32^.

Above we omitted the NV center’s own ^14^N (or ^15^N) nuclear spin: we assume that it is 100% polarized in the state with a given *m*_*I*_, and that the driving is applied at the frequency of the corresponding transition. Without such polarization, the hyperfine levels which are not in resonance with the Rabi driving field are quickly decohered, since the on-site hyperfine coupling (2.15 MHz for ^14^N, and 3.03 MHz for ^15^N) is comparable to a typical driving strength, so that overall detection quality degrades (see Supplementary Information). Fortunately, polarization of the NV’s own nuclear spin can be easily achieved in different ways^12^,^15^,^33^,^34^,^35^, and incorporated in the experimental protocol.

Concluding, we presented a scheme for one-by-one detection of the nuclear spins weakly coupled to an electron spin (e.g. the NV center in diamond), using the rotary echo protocol. The key feature of this scheme is the ability to experimentally adjust, and thus greatly narrow, the width of the resonance peaks corresponding to the detected nuclear spins. When compared with the existing approaches, the scheme proposed here provides significant improvement in the selectivity of the nuclear spin detection, albeit being more demanding to the quality of experimental setup (see Supplementary Information) in terms of the fast switching of the driving phase and the timing precision (~0.5 ns, determined by the width of the resonant peak), and increased interrogation time (equal to 4*NT*, about 1 ms per run for parameters used in Fig. 3). Moreover, the scheme discussed here, and the considerations above, are not specific to the NV center, and can be applied to a wide range of other electron-nuclear systems, such as dopant atoms in semiconductors and other color centers, using the electron spins as probes for the surrounding nuclei.

## Methods

### Analytical Calculations

NV center is subjected to the static magnetic field parallel to its quantization axis, and to a strong driving rf field at the frequency of the transition between the two lowest sublevels, *m*_*NV*_ = 0 and *m*_*NV*_ = 1 ( 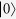 and 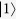, respectively), and coupled to a ^13^C nuclear spin *I* = 1/2 via dipole-dipole interaction; the sublevel *m*_*NV*_ = −1 is idle and thus ignored. Within the secular approximation, in the frame rotating with the frequency of the rf field, the system’s Hamiltonian is


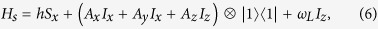


where 
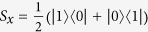
, *h* is the Rabi driving field, *ω*_*L*_ is the Larmor frequency of the nuclear spin, and *A*_*i*_ are the components of the hyperfine field created by the NV spin at the nuclear spin position. The coupling constants are 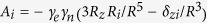, where *R* is the vector connecting NV center and the nuclear spin, *i* = *x*, *y*, *z*, *γ*_*e*_ and *γ*_*n*_ are the electronic and nuclear gyromagnetic ratios, and *δ*_*ij*_ is the Kronecker’s symbol. For a remote 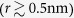 nucleus, the coupling strength 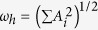 is small, so 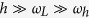. Introducing the operator 
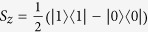
, with appropriate rotation of the nuclear spin coordinate axes, the Hamiltonian becomes


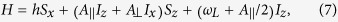


where 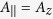 and 
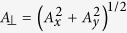
, and the terms 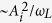 are neglected, since they only slightly renormalize *ω*_*L*_, 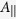, and 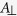. We parametrize 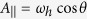 and 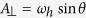, where *θ* is the angle between the hyperfine field and the NV symmetry axis, and is related to the polar angle, *θ*_*R*_, of the vector *R* through 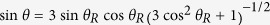 and 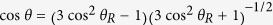.

The evolution operator *U* of a single rotary echo cycle is given by Eq. 2 of the main text. We analyze dynamics by finding ln*U* to the first order in the small parameter *g* = *ω*_*h*_/*h*. We achieve that by relating the Hamiltonian *H* of Eq. 1 and the non-interacting (zero-order) Hamiltonian 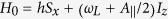 (see Eq. 3). This is done in two steps; first we apply to *H*_0_ the Schrieffer-Wolfe type transformation via the unitary operator 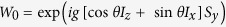, and obtain


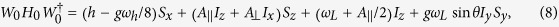


where we neglected terms of the order *g*^2^*ω*_*L*_, *g*^2^*ω*_*h*_, and higher. Next transformation, with 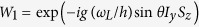, eliminates the last term in the equation above, and we find that 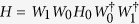. Correspondingly, for the evolution operator we have 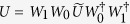, where


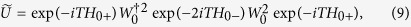


with *H*_0±_ = *H*_0_(±*h*); here *W*_1_ disappears as it commutes with *W*_0_ and is even with respect to the sign change of *h*. Expanding *W*_0_ in terms of small *g*, we find *U*, and re-write it as a single exponential,


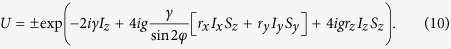


where *ϕ* = *hT*, and the coefficients *r*_*x*_ = −sin*θ*  cos*φ*  sin*ϕ*, *r*_*y*_ = sin*θ*  sin*φ  *cos*ϕ*, and *r*_*z*_ = −cos*θ*  sin*ϕ*; the total sign ± should be chosen to be the same as the sign of cos2*φ*, whereas the parameter 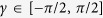 is given by tan*γ* = tan2*φ*. The rotary echo signal after *N* cycles is found by evaluating the trace, 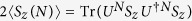; by writing *U* as a single exponential, *U*^*N*^ is evaluated in a straightforward but lengthy manner (see Supplementary Information for details), giving the results mentioned in the main text.

### Numerical Simulations

The numerical simulations were performed for one electron spin of the NV center, and 14 nuclear spins ^13^C located at the sites of the diamond lattice. To make the system reasonably realistic, the lattice sites for the first six nuclear spins were taken as close as possible to the spins detected in ref. 5. The rest of the spins were placed randomly on the diamond lattice, with the restriction that the resulting density of the ^13^C spins corresponds to their natural abundance of 1.08%. The simulations were performed in the rotating frame, using the Hamiltonian with 14 nuclear spins:


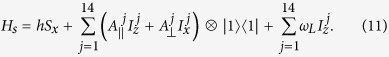


The electron spin of the NV center was assumed to be localized at the mid-point between the positions of the nitrogen atom and the vacancy. The hyperfine interaction between the *j*-th nuclear spin and the electron spin were assumed to be of purely dipole-dipole nature, and the interaction constants 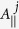 and 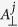 were calculated using the simulated locations of the nuclear spins. Additional numerical results and comparison with the pulse-based detection method are given in Supplementary Information.

## Additional Information

**How to cite this article**: Mkhitaryan, V. V. *et al.* Highly selective detection of individual nuclear spins with rotary echo on an electron spin probe. *Sci. Rep.* **5**, 15402; doi: 10.1038/srep15402 (2015).

## Supplementary Material

Supplementary Information
