# Supplementary material for: Highly selective detection of individual nuclear spins with rotary echo on an electron spin probe
Source: Sci Rep. 2015 Oct 26;5:15402. doi: 10.1038/srep15402 (PMC4620492; doi:10.1038/srep15402)
Supplement: Supplementary Information [file srep15402-s1.pdf]

Supplementary Information
Highly selective detection of individual nuclear spins with rotary echo on an electron spin probe

V. V. Mkhitaryan,[1] F. Jelezko,[2] and V. V. Dobrovitski[1]

[1]*Ames Laboratory US DOE, Ames, Iowa, 50011, USA*
[2]*University of Ulm, Institute of Quantum Optics and Center for Integrated Quantum Science and Technology, 89081 Ulm, Germany*

I. NUMERICAL RESULTS

A. Details of the simulations of the rotary-echo detection protocol

The numerical simulations were performed for one electron spin of the NV center, and 14 nuclear spins ^{13}C located at the sites of the diamond lattice. To make the system reasonably realistic, the first six nuclear spins were taken as close as possible to the spins detected in Ref.[1]. The rest of the spins were placed randomly, with the restriction that the resulting density of the ^{13}C spins corresponds to their natural abundance of 1.08%.

The simulations were performed in the rotating frame, using the Hamiltonian with $M = 14$ nuclear spins:

$$H_s = hS_x + \sum_{j=1}^{M} \left(A_{\|}^j I_z^j + A_{\perp}^j I_x^j\right) \otimes |1\rangle\langle 1| + \sum_{j=1}^{M} \omega_L I_z^j. \tag{1}$$

(cf. Eqs. 4, 5 and 6 below, and Eq. 1 of the main text). The electron spin of the NV center was assumed to be localized at the mid-point between the positions of the nitrogen atom and the vacancy. The hyperfine interactions between the j-th nuclear spins and the electron spin were assumed to be of purely dipole-dipole nature, so the coupling constants are (see also Sec. II A here for details):

$$A_{\|}^j = (\gamma_e \gamma_n / R_j^3)[1 - 3R_{jz}^2/R_j^2], \tag{2}$$

$$A_{\perp}^j = -3(\gamma_e \gamma_n / R_j^3) R_{jz} R_{j\perp} / R_j^2 \tag{3}$$

where $\mathbf{R_j}$ is the vector connecting the j-th nuclear spin and the NV electron spin, $R_{j\perp} = \sqrt{R_{jx}^2 + R_{jy}^2}$, γ_e and γ_n are the electronic and nuclear gyromagnetic ratios. The parameters $A_{\|}$, $A_{\perp}$, and ω_h of the nuclear spins, as well

Spin no.	$A_{\|}$	$A_{\perp}$	ω_h	T_3
1	0.47699	0.11761	0.49128	2.68018
2	0.24008	0.16038	0.28872	2.79253
3	0.22559	0.22788	0.32066	2.79857
4	-0.08334	0.09514	0.12648	2.96368
5	-0.14254	0.15641	0.21162	2.99632
6	0.08246	0.15047	0.17159	2.87315
7	0.24803	0.53965	0.59392	2.777
8	-0.12720	0.26725	0.29598	2.98505
9	-0.09858	0.16205	0.18968	2.97132
10	-0.12204	0.11479	0.16754	2.98527
11	0.03223	0.39732	0.39863	2.89312
12	-0.12673	0.04821	0.13559	2.98852
13	0.09115	0.05810	0.10809	2.86952
14	-0.08306	0.05549	0.09989	2.96385

TABLE I: Parameters of the nuclear spins used in the simulations. The hyperfine coupling constants $A_{\|}$ and $A_{\perp}$, and the values of ω_h are given in Mrad/s, while T_3 is in microseconds. The simulations assume external bias field of 400 G, which corresponds to the Larmor frequency of the ^{13}C nuclear spins of $\omega_L = 2.69131$ Mrad/s, or, equivalently, $\omega_L = 2\pi \cdot 428.336$ kHz.

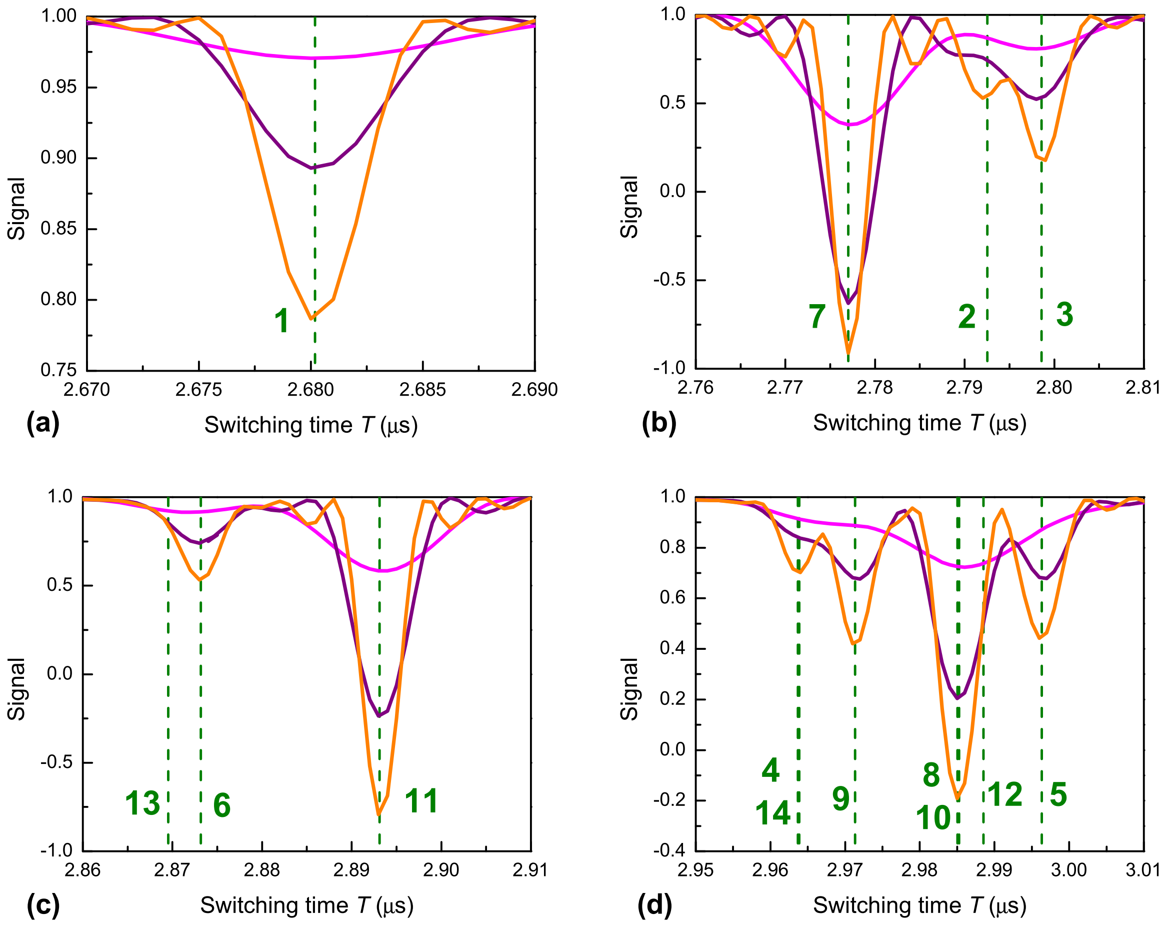

FIG. 1: (Color online) Rotary echo signals after $N = 30$ (magenta), 70 (purple), and 100 (orange) cycles, as a function of the switching time T for a NV spin coupled to 14 ^{13}C nuclear spins listed in the Table; $\omega_L = 2\pi \cdot 428$ kHz, and driving h is adjusted so that $hT = 28\pi$ (so that $4.59 \leq (h/2\pi) \leq 5.28$ MHz). The panels (a)–(d) show the regions corresponding to the resonances of the order $k = 3$ produced by different nuclear spins. Theoretically expected resonance positions are marked with green lines, the green numbers show which specific nuclear spin (as enumerated in the Table) produces a given resonance. Some resonances are produced by two spins located at the symmetry-related sites.

as the corresponding resonant switching times T_3 for the resonances of the order $k = 3$, are given in the Table as $T_3 = 5\pi/\sqrt{(2\omega_L + A_\parallel)^2 + A_\perp^2} \simeq 5\pi/[2\omega_L + A_\parallel]$ (in the first equality here, and in the Table, we took into account the second-order terms omitted in transition from Eq. 4 to Eq. 6 below, see also discussion in the Section II A below).

Note that the spins no. 4 and no. 14 are located at the symmetry-related sites, and have practically the same values of $A_\parallel$ (and, correspondingly, very close positions of the resonances). Another similar pair is the spins no. 8 and no. 10. In Fig. 3 of the main text, these resonances are marked with arrows.

In Fig. 1 we show how the resonance peaks develop with increasing the number of cycles. The peaks become sharper, and their magnitude increases; at even larger N some peaks become more shallow again, in accordance with the oscillatory nature of the function $L(N)$ in Eq. 5 of the main text and Eq. 16 here.

B. Comparison of the detection protocols based on the rotary echo and on the pulse dynamical decoupling

It is also interesting to look in more detail at the comparison of the rotary-echo based protocol with the detection based on pulse dynamical decoupling. The scheme which employs the pulsed dynamical decoupling[1–3], also produces resonances when the pulse delay τ is tuned to resonance with the nuclear spin rotation, $\tau_n = \pi(2n-1)/(2\omega_L + A_\parallel)$ with integer n, where ω_L is the Larmor frequency of the nuclear spins.

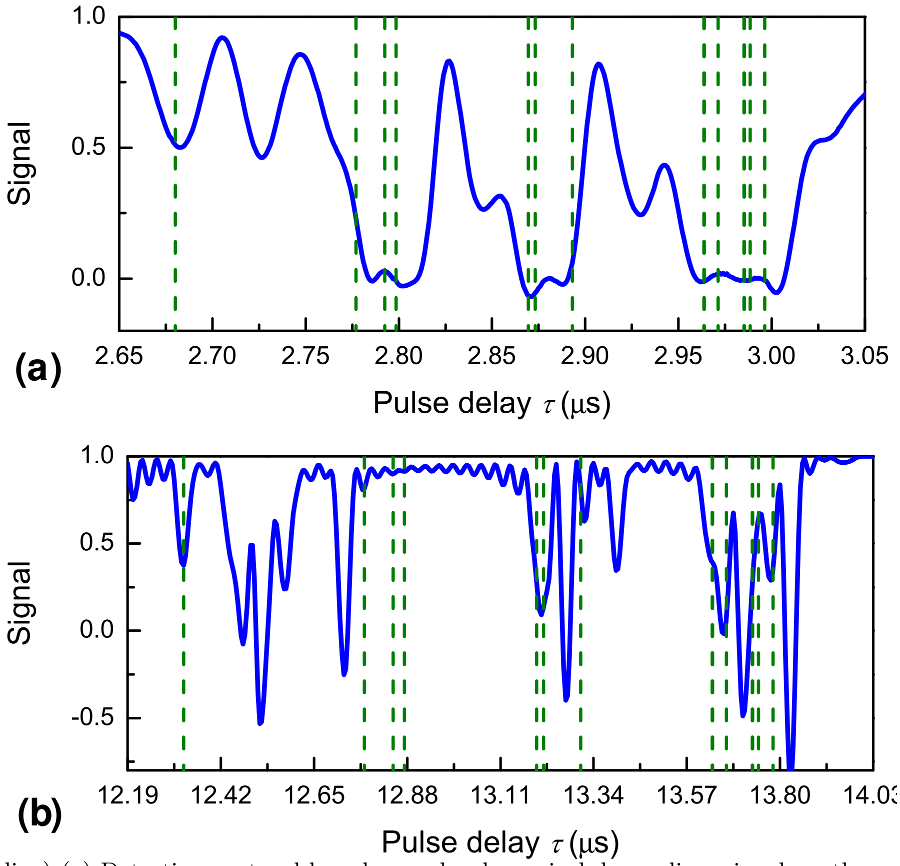

FIG. 2: (Color online) (a) Detection protocol based on pulse dynamical decoupling, signal vs. the pulse delay τ, for the same set of nuclear spins as shown in the Table, for 16 half-cycles of the XY4 sequence. The region corresponding to the resonances of the order $n = 3$ is shown. (b) Same as (a), but now the region of the resonances of the order $n = 12$. The resonances are much narrower (relative to their separation), but many peaks from the resonances of other orders fall within the region of interest.

For two nuclei with close hyperfine couplings ω_h^a and ω_h^b (where $\omega_h^{a,b} \sim \omega_h$, $|\omega_h^a - \omega_h^b| \ll \omega_h$), the distance between two resonances in this scheme is of order of $(2n-1)(\omega_h^a - \omega_h^b)/\omega_L^2$, while the width of each resonance is of order of ω_h/ω_L^2 (i.e., with increasing n the resonances from two spins become farther from each other, while their widths remain unchanged). Thus, the resonances are resolved when $(\omega_h^a - \omega_h^b)/\omega_h \gtrsim 1/(2n-1)$. When comparing the resonances of the same orders ($k = n$), the rotary-echo detection provides improvement in resolution by a factor of $\omega_L/h \sim 10$–20 for typical experimental parameters. This improvement is illustrated by comparison of the rotary echo protocol with the pulse decoupling: Fig. 2a shows the signal for the detection based on pulse dynamical decoupling for the same order of resonance $n = 3$, and where no well resolved peaks are seen.

To become comparable to the rotary-echo detection, one should use larger $n = 12$, the corresponding results are shown in Fig. 2b. Analysis of the resulting signal is more difficult in this case, because of the large number of peaks from the resonances of other orders which fall within the region of interest. Indeed, let us consider two spins a and b, with the corresponding resonant pulse delay times $\tau_n^j = \pi(2n-1)/(2\omega_L + A_\parallel^j)$, where $j = a, b$. With increasing n, the theoretical positions of the resonances, $\tau_n^j = \pi(2n-1)/(2\omega_L + A_\parallel^j)$, increase proportionally to n, i.e. the overall scale of the delay times uniformly stretches. Thus, the distance between the resonances of the *same order* produced by *different spins* increases with n: indeed, $\tau_n^b - \tau_n^a \propto n$. At the same time, the distance between resonances of *different orders* produced by the *same spin* remains unchanged: $\tau_{n+1}^j - \tau_n^j = 2\pi/(2\omega_L + A_\parallel)$. As a result, for larger orders n, the region of τ which contains the peaks of order n from all spins (i.e., the region from τ_n^a to τ_n^b), becomes wider as n increases. Correspondingly, the total number of peaks in this region increases, since more and more peaks of other orders ($\tau_{n\pm1}^j$, $\tau_{n\pm2}^j$, etc.) fall within the interval between τ_n^a to τ_n^b. As a result, when using higher-order resonances, we see a large number of extra peaks, created by the same spins but belonging to the resonances of other orders.

In order to compare the simulation results, note that the single, well-isolated peak corresponding to the spin no. 1

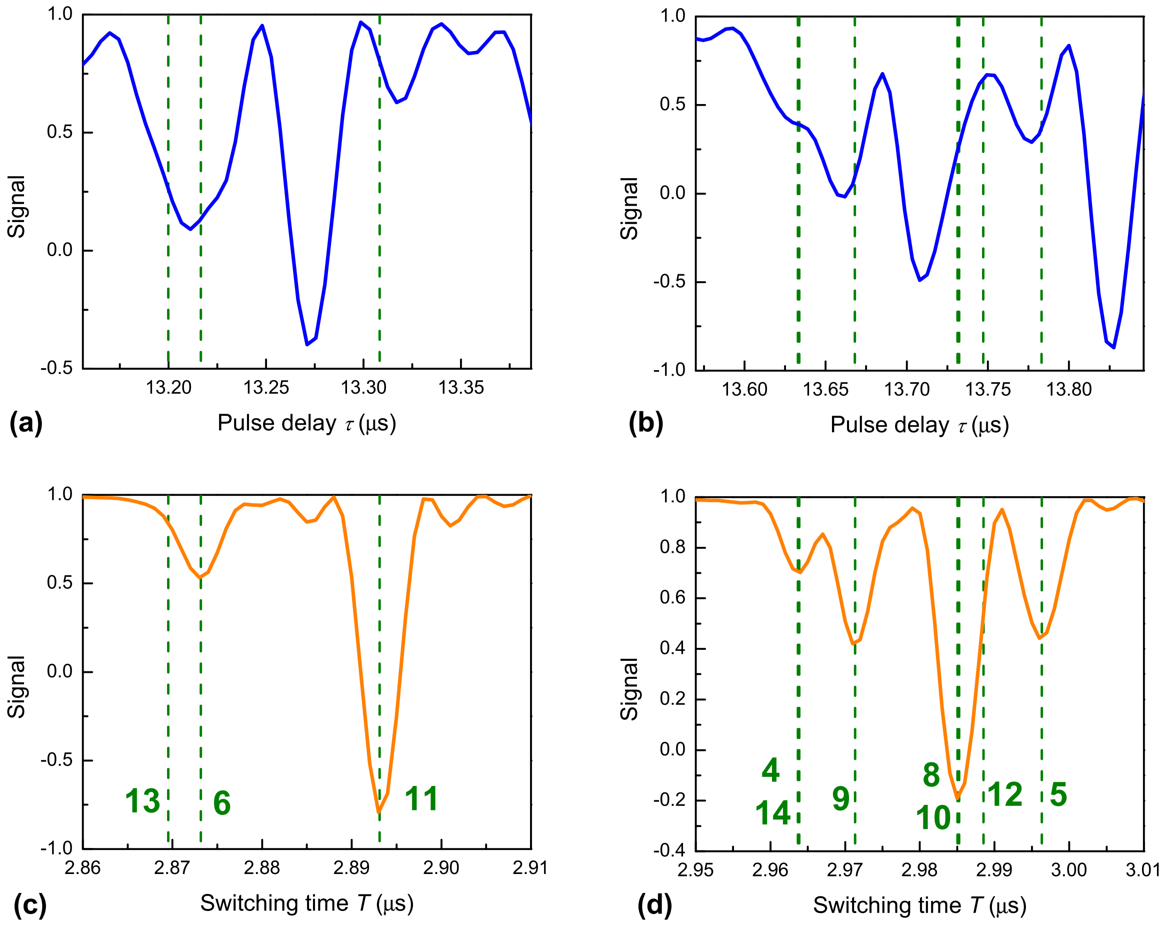

FIG. 3: (Color online) Comparison of the detection schemes based on the pulse decoupling protocol [(a) and (b), blue lines] with $n = 12$, and the rotary echo protocol [(c) and (d), orange lines] with $k = 3$. The regions shown correspond to the spins nos. 13, 6, and 11 [(a) and (c)], and nos. 4, 14, 9, 8, 10, 12, and 5 [(b) and (d)]. Theoretically expected resonance positions are marked with green lines, the green numbers show which specific nuclear spin (as enumerated in the Table) produces a given resonance.

(the leftmost peak in Fig. 2) is resolved well by both methods. Next, the peaks corresponding to the spins nos. 2, 3, and 7 are not present in Fig. 2 at all, and longer/shorter interrogation times of the pulse decoupling protocol are needed to see them. Thus, we focus on the comparison of the regions corresponding to the spins nos. 13, 6, and 11, and nos. 4, 14, 9, 8, 10, 12, and 5; these regions are shown in Fig. 3. The results show the advantages of the rotary echo protocol (besides the absence of many extra peaks): although not perfect, the peaks are more pronounced, not shifted away from their theoretically expected positions, and overall representation of the resonances is greatly improved.

The rotary echo-based scheme proposed in this work provides significant improvement in the selectivity of the nuclear spin detection, albeit being more demanding to the quality of experimental setup. One issue is the total interrogation time for large $N = 50$–100: e.g., for a single experimental run consisting of 100 cycles, each of duration 4×3 μs (see the typical scale for T in any figure above), the total time is about 1.2 ms; typically 10^5–10^6 runs are needed for good measurement of the signal (to suppress the shot noise), which means that a single data point requires at least a few minutes, and the total interrogation time for the resonance curve would be hours. Another requirement is that throughout each run the driving field should be switched very fast, and the timing precision should be at the level of ~ 0.5 ns or better (determined by the width of the resonance peak).

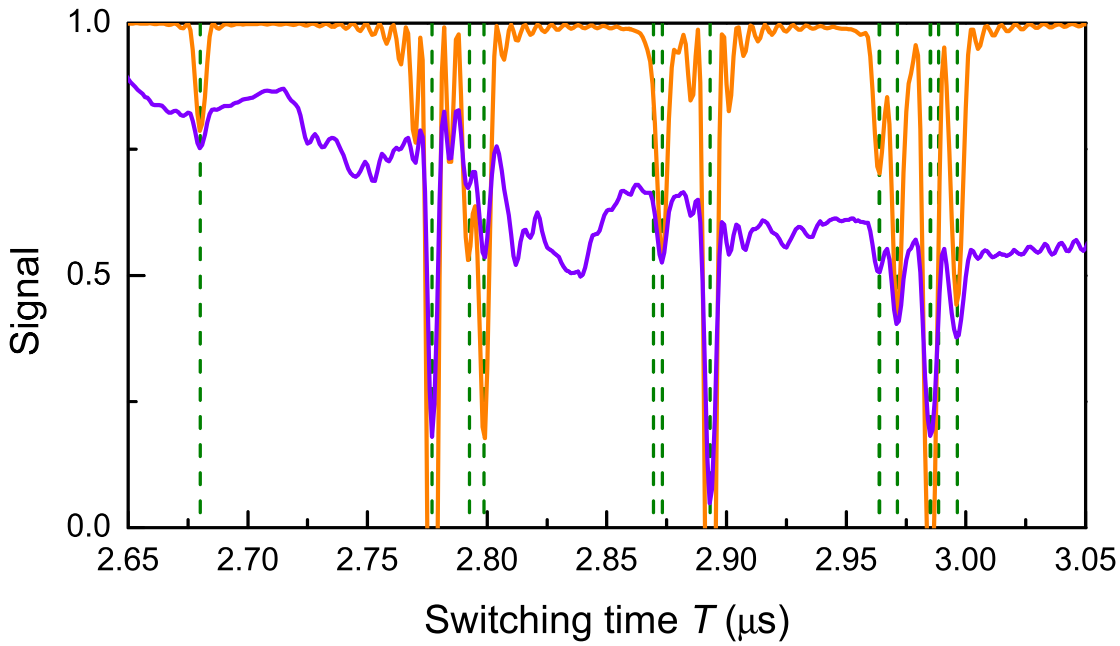

FIG. 4: (Color online) Comparison of the rotary-echo detection with polarized (orange) and unpolarized (violet) spin of the NV's own ^{14}N nuclear spin.

C. Effect of the NV's own nuclear spin

Finally, we discuss the role of the NV's own nuclear spin, assuming the natural ^{14}N nucleus with $I = 1$ and the hyperfine coupling to the NV's electronic spin of $A_0 = 2.16\ MHz$. Under the experimental conditions considered throughout the paper, the hyperfine interaction Hamiltonian has the diagonal form $H_{\rm hf} = A_0 S_z I_z$. In its natural state the ^{14}N spin is unpolarized, and its density matrix is a uniform incoherent mixture of the states $m_I = 0, \pm 1$. In this case, the NV electronic spin evolves as if it were subjected to an additional static field with the magnitude randomly chosen from 0, +2.16 MHz, and −2.16 MHz. If the ^{14}N is polarized, e.g. in the state $m_I = 0$, its impact is null.

Fig. 4 shows the simulations performed for the unpolarized ^{14}N spin, and compares the results to the ^{14}N spin polarized in the state $m_I = 0$ (as is the case for all other simulations shown above and in the main text). Without polarization, the overall detection quality degrades. Many peaks still remain well pronounced, but the overall reduction of the signal, appearance of extra wiggles, and the decreased visibility of smaller peaks create problems for accurate detection. Thus, although the rotary-echo protocol still works with unpolarized ^{14}N spin, it is desirable to polarize the NV's own nuclear spin for detection. Fortunately, this step is not difficult, and can be achieved in many different ways.

II. ANALYTICAL DERIVATION OF THE EVOLUTION OPERATOR AND OF THE ELECTRONIC SPIN

A. Interaction Hamiltonian

We consider NV electronic ground state in the static magnetic field antiparallel to its quantization axis, subject to a strong driving rf field at the frequency of the transition between the two lowest-lying sublevels, $m_{NV} = 0$ and $m_{NV} = 1$ ($|0\rangle$ and $|1\rangle$, respectively), and coupled to a ^{13}C nuclear spin $I = 1/2$ with a magnetic dipole-dipole interaction. The NV centers sublevel $m_{NV} = -1$ is practically idle and can be ignored. Within the secular approximation and in the frame rotating with the frequency of the rf field, this system is described by the Hamiltonian,

$$H_s = hS_x + \big(A_x I_x + A_y I_x + A_z I_z\big) \otimes |1\rangle\langle 1| + \omega_L I_z, \tag{4}$$

where $S_x = \frac{1}{2}\big(|1\rangle\langle 0| + |0\rangle\langle 1|\big)$, h is the Rabi driving field, ω_L is the Larmor frequency of the nuclear spin, and A_i are the components of the hyperfine field created by the NV spin at the nuclear spin position. Introducing the radius-vector connecting the NV center with the nuclear spin, $\mathbf{R}$, we have $A_i = \gamma_e\gamma_n(3R_zR_i/R^5 - \delta_{zi}/R^3)$, where $i = x, y, z$, γ_e and γ_n are the electronic and nuclear gyromagnetic ratios, and δ_{ij} is the Kronecker's symbol. We consider a remote ($r \gtrsim 0.5$ nm) nuclear spin so that the hyperfine coupling strength, $\omega_h = (\sum A_i^2)^{1/2}$, is the smallest parameter in the problem, and we have $h \gg \omega_L \gg \omega_h$. It is further convenient to introduce the NV spin operator, $S_z = \frac{1}{2}\big(|1\rangle\langle 1| - |0\rangle\langle 0|\big)$, and rotate the coordinate frame of the nuclear spin in the $x-y$ plane, re-writing the Hamiltonian as

$$H_s = hS_x + S_z\big(A_\parallel I_z + A_\perp I_x\big) + (\omega_L + A_\parallel/2)I_z + (A_\perp/2)I_x, \tag{5}$$

where $A_\parallel = A_z$ and $A_\perp = (A_x^2 + A_y^2)^{1/2}$. After another rotation of the nuclear spin coordinate axes in the $x-z$ plane, the Hamiltonian (4) is transformed to a standard form

$$H = hS_x + \big(A_\parallel I_z + A_\perp I_x\big)S_z + (\omega_L + A_\parallel/2)I_z, \tag{6}$$

where we neglected terms $\sim A_i^2/\omega_L$, which would otherwise slightly renormalize ω_L, $A_\parallel$, and $A_\perp$. This renormalization, for instance, replaces the last term above, $(\omega_L + A_\parallel/2)I_z$, by a slightly more complicated expression $\sqrt{(\omega_L + A_\parallel/2)^2 + (A_\perp/2)^2 I_z}$; this slightly changes the resonant switching times T_k, and that change is taken into account in the Table above.

Furthermore, we parametrize $A_\parallel = \omega_h \cos\theta$ and $A_\perp = \omega_h \sin\theta$, where θ is the angle between the hyperfine field and the NV symmetry axis. It is related to the polar angle, θ_R, of the radius-vector $\mathbf{R}$ through $\sin\theta = 3\sin\theta_R\cos\theta_R(3\cos^2\theta_R + 1)^{-1/2}$ and $\cos\theta = (3\cos^2\theta_R - 1)(3\cos^2\theta_R + 1)^{-1/2}$.

B. Rotary echo cycle

The evolution operator of single rotary echo cycle is given by

$$U \approx U_0 = \exp(-iTH_+)\exp(-2iTH_-)\exp(-iTH_+). \tag{7}$$

where T is the half-period of the rf amplitude reversal, and $H_\pm = H(\pm h)$. We are interested in the regime of strong driving, $h \gg \omega_L$, ω_h, where the first term in Eq. (6) is strongly dominant. To gain a qualitatively clear picture of what behavior of rotary echoes is expected, we note that the Hamiltonian (6) nearly commutes with $H_0 = hS_x + (\omega_L + A_\parallel/2)I_z$, whereas the hyperfine coupling mixes the eigenvectors of H_0 only slightly, $\sim \omega_h/h$. Hence the evolution operator is approximately given by

$$U \approx \exp\Big[-i4T\big(\omega_L + A_\parallel/2\big)I_z\Big] = \cos 2\varphi - 2iI_z \sin 2\varphi, \tag{8}$$

where $\varphi = (\omega_L + A_\parallel/2)T$. It describes a nuclear spin perfectly decoupled from the NV center spin. On the other hand, from Eq. (8) it follows that in the narrow region $|\sin 2\varphi| \lesssim \omega_h/h$ the evolution operator U approaches unity, and the mixing caused by the hyperfine interaction becomes important. Thus the approximation Eq. (8) fails around $\sin 2\varphi = 0$, where the NV center and target nuclear spins become entangled resonantly.

In the following we analyze this resonant entanglement quantitatively, in the regime $h \gg \omega_L, \omega_h$, by finding $\ln U$ to the first order in the small parameter $g = \omega_h/h$. This can be done by relating H_0 and H through a unitary transformation. We apply to H_0 a small transformation with the operator, $W_0 = \exp\big(ig[\cos\theta I_z + \sin\theta I_x]S_y\big)$. To the first order in g this transformation results in

$$W_0 H_0 W_0^\dagger = \big(h - g\omega_h/8\big)S_x + \big(A_\parallel I_z + A_\perp I_x\big)S_z + (\omega_L + A_\parallel/2)I_z + g\omega_L \sin\theta I_y S_y, \tag{9}$$

where we kept the necessary accuracy by neglecting terms $\sim g^2\omega_L$, $g^2\omega_h$ and higher. The right hand side of Eq. (9) differs from H by the renormalization of h and the presence of the last term. Another, even smaller transformation with $W_1 = \exp\big(-ig(\omega_L/h)\sin\theta I_y S_z\big)$ eliminates this term; within the desired accuracy and neglecting the small renormalization of h we get:

$$W_1 W_0 H_0 W_0^\dagger W_1^\dagger = H. \tag{10}$$

Thus for the evolution operator we have $U = W_1 W_0 \tilde{U} W_0^\dagger W_1^\dagger$, where

$$\tilde{U} = \exp(-iTH_{0+})W_0^{\dagger 2}\exp(-2iTH_{0-})W_0^2\exp(-iTH_{0+}), \tag{11}$$

with $H_{0\pm} = H_0(\pm h)$. In this relation, W_1 disappears as it commutes with W_0 and is even with respect to the sign change of h. It is now straightforward to expand W_0 in Eq. (11) over small g and find U to the first order in g:

$$U = \exp(-4i\varphi I_z) + 4ig\big[r_x I_x S_z + r_y I_y S_y + r_z \cos 2\varphi I_z S_z\big] + 2gr_z \sin 2\varphi S_z + \mathcal{O}(g^2), \tag{12}$$

where we have introduced the angle, $\phi = hT$, and the dimensionless coefficients,

$$r_x = -\sin\theta\cos\varphi\sin\phi, \quad r_y = \sin\theta\sin\varphi\cos\phi, \quad r_z = -\cos\theta\sin\phi. \tag{13}$$

We are going to find the rotary echo signal after N cycles by evaluating the trace, $2\langle S_z(N)\rangle = \mathrm{Tr}\big(U^N S_z U^{\dagger N} S_z\big)$. To this end we rewrite the evolution operator (12) in terms of a single exponent:

$$U = \pm\exp\left(-2i\gamma I_z + 4ig\frac{\gamma}{\sin 2\varphi}\big[r_x I_x S_z + r_y I_y S_y\big] + 4igr_z I_z S_z\right). \tag{14}$$

Here the total sign is defined as $\pm \equiv \mathrm{sign}(\cos 2\varphi)$, whereas the parameter $\gamma \in [-\pi/2, \pi/2]$ is given by $\tan\gamma = \tan 2\varphi$. Derivation of Eq. (14) is based on the mathematical formula $\exp(-A)\partial_g \exp(A + gB)|_{g=0} = f(\mathrm{ad}A)(B)$, where A and B are operators, $f(z) = (1 - e^{-z})/z$, and $\mathrm{ad}A(B) = [A, B]$. Further evaluation of $\mathrm{Tr}\big(U^N S_z U^{\dagger N} S_z\big)$ is based on the fact that $U^N = \exp(i\hat{M})$, where the operator $\hat{M} = -2\Gamma I_z + 4(\Lambda_x I_x + \Lambda_z I_z)S_z + 4\Lambda_y I_y S_y$ commutes with $\hat{P} = -4\Gamma\Lambda_z S_z + 8\Lambda_y(\Lambda_x I_z - \Lambda_z I_x)S_x$. Introducing the scalars, $\Omega^2 = \Gamma^2 + \Lambda_x^2 + \Lambda_y^2 + \Lambda_z^2$, $\Pi^2 = \hat{P}^2 = 4\Gamma^2\Lambda_z^2 + 4\Lambda_y^2(\Lambda_x^2 + \Lambda_z^2)$, $2C_1 = \sqrt{\Omega^2 + \Pi} + \sqrt{\Omega^2 - \Pi}$, and $2\Pi C_2 = \sqrt{\Omega^2 + \Pi} - \sqrt{\Omega^2 - \Pi}$, we can write the exponent as

$$\exp(i\hat{M}) = \cos C_1 \cos \Pi C_2 - \hat{P}\sin C_1 \sin \Pi C_2 + i\hat{M}\frac{C_1 - C_2\hat{P}}{C_1^2 - C_2^2\Pi^2}\big(\sin C_1 \cos \Pi C_2 + \hat{P}\cos C_1 \sin \Pi C_2\big). \tag{15}$$

After a lengthy but straightforward calculation we find:

$$\langle S_z(N)\rangle = \frac{1}{2}\mathrm{Tr}\big(U^N S_z U^{\dagger N} S_z\big) = \frac{1}{2} - \frac{g^2\mu^2}{\sin^2 2\varphi + g^2\mu^2}L(N), \tag{16}$$

where $\mu^2 = \sin^2\theta\cos^2\phi\big(\sin^2\varphi + \frac{1}{4}\gamma^2\tan^2\theta\big)$, and

$$L(N) = \frac{1 - \cos N(\Omega_+ + \Omega_-)}{2} - \left[(\Omega_+ + \Omega_-)^2 - (2\gamma\sin\varphi\sin\theta\cos\phi)^2\frac{\sin^2\gamma + g^2\mu^2}{\mu^2\sin^2\gamma}\right]\frac{\sin N\Omega_+ \sin N\Omega_-}{4\Omega_+\Omega_-}, \tag{17}$$

with

$$\Omega_\pm = \sqrt{\gamma^2 + g^2\left[\frac{\gamma^2}{\sin^2\gamma}(r_x^2 + r_y^2) + r_z^2\right] \pm 2g\sqrt{\gamma^2 r_z^2 + g^2\frac{\gamma^2}{\sin^2\gamma}r_y^2\left[\frac{\gamma^2}{\sin^2\gamma}r_x^2 + r_z^2\right]}}. \tag{18}$$

We refrain from very small values of $A_\parallel$ and put $\cos\theta \gg g$; then μ is a restricted quantity. So is $L(N)$, which varies between 0 and 1. Hence, Eq. 16 clearly describes a resonance behavior. Indeed, the prefactor of $L(N)$ in Eq. (16) is suppressed as g^2, unless $|\sin 2\varphi|$ is smaller than $g\mu$, where it abruptly grows to 1. This occurs at vanishing $\cos\varphi \to 0$. In contrast, at $\sin\varphi \to 0$ the resonance is excluded, because in the latter case μ and $L(N)$ also go to zero. Near the resonance one has $\gamma \simeq -\sin 2\varphi \to 0$, $\Omega_\pm \simeq g|r_z \pm r_y|$, and $L(N) \simeq \big[1 - \cos N(\Omega_+ + \Omega_-)\big]/2$, i.e. for $N \gg h/A_\perp$ it represents fast modulation inside the envelope given by the prefactor. Therefore, for large N the resonance is characterized by the width, $\Delta\varphi = g\sin\theta\cos\phi$, and depth, $\sin^2(gN\sin\theta\cos\phi)$, with well resolved peaks at larger N. We finalize our consideration of the NV electron coupled to a single target nuclear spin by noting that accidental coincidence of $\cos\varphi = 0$ and $\cos\phi = 0$ destroys the resonance.

Note that at the resonance, if the driving h is chosen in an optimal manner, to ensure $\phi = hT = \pi m$ with integer m, the coupling between the nuclear spin and the NV spin is implemented by the term $I_y S_y$ in the evolution operator (14), because $r_x = r_z = 0$ under these conditions. Interaction between the unpolarized nuclear spin (whose density matrix is proportional to identity matrix) and the NV spin leads to decoherence of the latter in the basis $|\pm\rangle$, where $|\pm\rangle$ are the eigenstates of the operator S_y with the eigenvalues $\pm 1/2$ respectively. This decoherence leads to decay of the measured S_z component of the NV spin.

In a realistic situation the NV center spin is coupled to many nuclear spins. Ignoring interactions between the nuclear spins, the Hamiltonian of the system is given by

$$H = hS_x + \sum_{j=1}^{M}\big(A_\parallel^j I_z^j + A_\perp^j I_x^j\big)S_z + \sum_{j=1}^{M}\big(\omega_L + A_\parallel^j/2\big)I_z^j. \tag{19}$$

Unlike the detection schemes based on the multipulse decoupling of the electron spin, the time evolution operator of rotary echo cycle in the presence of multiple nuclear spins is not a simple product of evolution operators for individual nuclear spins Eq. (14), because of the presence of external NV spin driving. In other words, the driving leads to the effective NV-mediated intrabath interaction, which greatly complicates the quantitative analysis of the rotary echo time evolution. Further progress can be made provided that the influence of the entire bath on the NV spin is smaller than the external driving, $\left|\sum_j (A_\parallel^j I_z^j + A_\perp^j I_x^j)\right| \ll h$, a plausible condition for an unpolarized bath. Assuming this is the case, the generalized transformation operator $W_0 = \exp\left(i\sum_j g_j[\cos\theta_j I_z^j + \sin\theta_j I_x^j]S_y\right)$, where $g_j = \omega_h^j$, $\tan\theta_j = A_\perp^j/A_\parallel^j$, is still close to unity. A generalization of Eq. (10) is now achieved with $H_0 = hS_x + \sum_j(\omega_L + A_\parallel^j/2)I_z^j$, and $W_1 = \exp\left(-i(\omega_L/h)\sum_j g_j \sin\theta_j I_y^j S_z\right)$: neglecting terms $\sim \omega_h g^2$, $\omega_L g^2$ and higher, we get:

$$W_1 W_0 H_0 W_0^\dagger W_1^\dagger = h\left(1 - \frac{1}{2h^2}\left[\sum_{j=1}^{M}(A_\parallel^j I_z^j + A_\perp^j I_x^j)\right]^2\right)S_x + \sum_{j=1}^{M}(A_\parallel^j I_z^j + A_\perp^j I_x^j)S_z + \sum_{j=1}^{M}(\omega_L + A_\parallel^j/2)I_z^j, \tag{20}$$

Formally, the right hand side of this equation differs from the Hamiltonian (19) by the extra coupling between S_x and the nuclear spin bath. We neglect this coupling for two reasons. First, according to our above assumption of an unpolarized bath, it is subleading to Eq. (19). Second, the rotary echo protocol suppresses its effect in the leading order, as it is an odd function of h. Hence we equate the right hand side of Eq. (20) to the Hamiltonian Eq. (19), thus generalizing Eq. (10). Subsequently, Eq. (12) generalizes to

$$U = \exp\left(-4i\sum \varphi_j I_z^j\right)\left(1 + 4i\sum_k g_k\left(r_z^k I_z^k S_z + \exp(4i\varphi_k I_z^k)\left[r_x^k I_x^k S_z + r_y^k I_y^k S_y\right]\right) + \mathcal{O}(g^2)\right), \tag{21}$$

where $\varphi_j = (\omega_L + A_\parallel^j/2)T$, and $r_{x,y,z}^j$ are given by Eq. (13) with $\phi = hT$ and individual θ_j. Up to an inessential overall sign, this still can be rewritten in terms of the exponent,

$$U = \exp\sum_j\left(-2i\gamma_j I_z^j + 4ig_j\frac{\gamma_j}{\sin 2\varphi_j}\left[r_x^j I_x^j S_z + r_y^j I_y^j S_y\right] + 4ig_j r_z^j I_z^j S_z\right), \tag{22}$$

where γ_j are defined in full analogy with the corresponding quantity in Eq. (14).

Unfortunately, further steps allowing to evaluate the trace, $\mathrm{Tr}(U^N S_z U^{\dagger N} S_z)$, starting from Eq. (22), do not seem to be workable analytically. The reason is the trace can not be easily represented as a product of traces over the individual nuclear spins, as different components of $\mathbf{S}$ do not commute. This luck of factorizability presumably means that, for general values of parameters, the NV driving-mediated intrabath interactions lead to the interfering resonance dips. It is however not the case with the optimal choice of driving amplitude, $hT = \pi m$, which ensures $\sin\phi = 0$. As follows from Eq. (13), in this case one has vanishing $r_x^j = r_z^j = 0$. Then the N cycle evolution operator U^N factors out into the product of similar evolution operators of individual nuclear spins, and $2\langle S_z(N)\rangle$ becomes the product of the doubled right hand side of Eq. (16) over the nuclear spin bath. In turn, the latter equation simplifies, leading to the answer

$$\langle S_z(N)\rangle = \frac{1}{2}\prod_j\left(1 - \frac{2g_j^2\mu_j^2}{\sin^2 2\varphi_j + g_j^2\mu_j^2}L_j(N)\right), \tag{23}$$

where $\mu_j^2 = \sin^2\theta_j\left(\sin^2\varphi_j + \frac{1}{4}\gamma_j^2\tan^2\theta_j\right)$, and

$$L_j(N) = \sin^2\varphi_j\sin^2\theta_j\frac{\sin^2\gamma_j + g_j^2\mu_j^2}{\mu_j^2(\sin^2\gamma_j + g_j^2\sin^2\varphi_j\sin^2\theta_j)}\sin^2 N\Omega_j, \qquad \Omega_j = \sqrt{\gamma_j^2 + \frac{g_j^2\gamma_j^2}{\sin^2\gamma_j}\sin^2\varphi_j\sin^2\theta_j}. \tag{24}$$

The NV electron spin resonates with m-th nuclear spin when $\cos\varphi_m \to 0$, and hence $\gamma_m \to 0$, $|\sin\varphi_m| \to 1$. Then the resonance dip has a width, $\Delta\varphi_m = g_m\sin\theta_m$, and depth, $L_m(N) = \sin^2(Ng_m\sin\theta_m)$.

Note that by optimizing the driving strength h such that $\phi = hT = \pi m$, one achieves several goals at once. First, the amplitude of the peak is maximized. Second, the interference between different spins is minimized. Third, the sensitivity to slow fluctuations of the driving power is minimized, since the derivative of $\cos\phi$ is zero for this optimal

driving: this is an addition to the fact that the rotary echo protocol, due to periodic switching of the driving phase, is inherently robust to fluctuations in the driving.

[1] T. H. Taminiau, J. J. T. Wagenaar, T. van der Sar, F. Jelezko, V. V. Dobrovitski, and R. Hanson, Phys. Rev. Lett. **109**, 137602 (2012).

[2] S. Kolkowitz, Q. P. Unterreithmeier, S. D. Bennett, and M. D. Lukin, Phys. Rev. Lett. **109**, 137601 (2012).

[3] N. Zhao, J. Honert, B. Schmid, M. Klas, J. Isoya, M. Markham, D. Twitchen, F. Jelezko, R.-B. Liu, H. Fedder, and J. Wrachtrup, Nature Nanotech. **7**, 657 (2012).
